# Inferring lncRNA Functional Similarity Based on Integrating Heterogeneous Network Data

**DOI:** 10.3389/fbioe.2020.00027

**Published:** 2020-02-06

**Authors:** Jianwei Li, Yingshu Zhao, Siyuan Zhou, Yuan Zhou, Liying Lang

**Affiliations:** ^1^Institute of Computational Medicine, School of Artificial Intelligence, Hebei University of Technology, Tianjin, China; ^2^MOE Key Lab of Cardiovascular Sciences, Department of Biomedical Informatics, Center for Noncoding RNA Medicine, School of Basic Medical Sciences, Peking University, Beijing, China

**Keywords:** lncRNAs, miRNAs, expression profiles, mRNAs, lncRNA functional similarity, integrated heterogeneous network data, web server

## Abstract

Although lncRNAs lack the potential to be translated into proteins directly, their complicated and diversiform functions make them as a window into decoding the mechanisms of human physiological activities. Accumulating experiment studies have identified associations between lncRNA dysfunction and many important complex diseases. However, known experimentally confirmed lncRNA functions are still very limited. It is urgent to build effective computational models for rapid predicting of unknown lncRNA functions on a large scale. To this end, valid similarity measure between known and unknown lncRNAs plays a vital role. In this paper, an original model was developed to calculate functional similarities between lncRNAs by integrating heterogeneous network data. In this model, a novel integrated network was constructed based on the data of four single lncRNA functional similarity networks (miRNA-based similarity network, disease-based similarity network, GTEx expression-based network and NONCODE expression-based network). Using the lncRNA pairs that share the target mRNAs as the benchmark, the results show that this integrated network is more effective than any single networks with an AUC of 0.736 in the cross validation, while the AUC of four single networks were 0.703, 0.733, 0.611, and 0.602. To implement our model, a web server named IHNLncSim was constructed for inferring lncRNA functional similarity based on integrating heterogeneous network data. Moreover, the modules of network visualization and disease-based lncRNA function enrichment analysis were added into IHNLncSim. It is anticipated that IHNLncSim could be an effective bioinformatics tool for the researches of lncRNA regulation function studies. IHNLncSim is freely available at http://www.lirmed.com/ihnlncsim.

## Introduction

Long non-coding RNAs (lncRNAs) are a class of RNA abundant in the transcriptome of eukaryotes with exceeding 200 nucleotides (Mercer et al., 2009). Although lncRNAs are not equipped with complete open reading frame, they could interfere with downstream gene expression via base complementary pairing, and play key regulatory roles in almost every important life activity, including transcriptional regulation, epigenetic gene regulation, post-transcriptional control, protein activity regulation, and the like (Ponting et al., 2009; Geisler and Coller, 2013). For example, lncRNAs can act as decoys of RNA-binding proteins or miRNAs to promote or inhibit the translation of target mRNAs through the base-pairing (Yoon et al., 2013). More importantly, lncRNAs also exhibit significant abnormal behaviors in the development of some complex diseases like cancers (Kong et al., 2014; Shi et al., 2014; Yao et al., 2014) and cardiovascular diseases (Congrains et al., 2012). For example, mechanistic investigations showed that lncRNA MALAT1 in renal cell carcinoma was over-expressed and MALAT1 could emerge as a new gene regulator or prognostic marker (Hirata et al., 2015). Additionally, lncRNA NEAT11 was identified as an oncogenic gene in non-small cell lung cancer and acted as a competing endogenous RNA of miR-377-3p to antagonize this miRNA function (Sun et al., 2016). The emergence of these recent research results has provided new ideas for some complex disease diagnosis, treatment and prognosis at lncRNA level.

As high-throughput sequencing technology was gradually mature, a number of lncRNA-related databases (Yotsukura et al., 2017) had been established for different purposes. Nowadays, how to verify the complex lncRNA function mechanism by various computational methods has become a research hotspot in the field of lncRNA regulation function studies and understanding of complex disease mechanism. Although the functions of some lncRNAs had been deeply explored in previous experimental verifications, confirmed lncRNA functions are still very limited. On the other hand, computational methods for lncRNA function predictions had benefited both disease biomarker detection and drug discovery. Furtherly, most available computational lncRNA function prediction methods heavily relied on the reasonable measurement of the similarities between functionally known and unknown lncRNAs. In addition, lncRNA functional similarity network data was normally applied to other algorithms like the prediction of lncRNA-disease associations. These computational models can be a screening tool for biological experiments, which would promote experimental efficiency for identifying the potential function of lncRNAs in diseases. The models of lncRNA similarity calculation, which were developed in previous studies, could be mainly divided into three categories according to their theoretic foundations. In order to state the different algorithms comprehensively, we had established a table with their brief descriptions (see Table 1).

**TABLE 1:** Categories and corresponding models of lncRNA functional similarity calculation.

		**Output**	
**Input data**	**Models**	**data**	**Description**
lncRNA interactions	IntNetLncSim, LFSCM	Values of lncRNA functional similarity	IntNetLncSim integrated four RNA interaction network data to represent lncRNAs as vectors, LFSCM integrated lncRNA-miRNA interaction and miRNA functional similarity information
lncRNA-disease association	LNCSIM, ILNCSIM, FMLNCSIM	Values of lncRNA functional similarity	These three models were all based on the lncRNA-disease association information and the directed acyclic graph structure of disease terminology
lncRNA expression profiles	IRWRLDA, PLNRGO	Values of lncRNA functional similarity	IRWRLDA utilized spearman correlation coefficient analysis between lncRNA expression profiles, PLNRGO exploited the Pearson correlation coefficient between lncRNA expression profiles
All of above data	IHNLncSim (Our method)	Values of lncRNA functional similarity, Graphical visualization	IHNLncSim exploited the AUC of four single networks as the weight and calculated the weighted average of miRNA-based lncRNA similarity network data, disease-based lncRNA similarity network data and two expression-based network data.

The first category of the models depended on lncRNA target gene information, which were built based on the principle that similar lncRNAs can interact with the similar sets of mRNAs and/or miRNAs. For example, IntNetLncSim (Cheng et al., 2016) integrated the data of four networks, which were lncRNA-mRNA interaction network, lncRNA-miRNA interaction network, miRNA-mRNA interaction network and mRNA-mRNA interaction network. In this model, each lncRNA may be represented by a vector of weights which was related to target miRNAs/mRNAs of the lncRNA. Another model named LFSCM was developed by Chen (2015). It was established by following three steps: (1) calculating disease semantic similarity based on MeSH; (2) calculating miRNA functional similarity based on disease semantic similarity and miRNA-disease associations; (3) calculating lncRNA functional similarity based on both miRNA functional similarity and lncRNA-miRNA interactions. The second category of models adopted the information of lncRNA-disease association as the key feature, which was based on the assumption that functionally similar lncRNAs tended to the same regulating functions in the same kind of disease. Chen et al. (2015, 2016a) and Huang et al. (2016) developed the models of LNCSIM, ILNCSIM and FMLNCSIM for measuring lncRNA functional similarity successively. These models were all based on the directed acyclic graph structure of disease terminology, which described the semantic similarity of diseases with the same node positions and quantity information, and then lncRNA similarity scores were calculated. Finally, it is well known that lncRNAs with similar expression patterns may have similar regulating functions, so the third category models gather similarities among the lncRNA expression profiles. For example, spearman correlation coefficient analysis was utilized to compute the lncRNA expression similarity between the expression profiles of each lncRNA pair in the model of IRWRLDA (Chen et al., 2016b). Deng et al. (2018) exploited the Pearson correlation coefficient between lncRNA expression profiles as lncRNA functional similarity.

Through analyzing the characters of these models carefully, most of the models had been developed with the data from single category, which only described lncRNA functional similarity in one aspect. More experiments showed that lncRNAs exhibit high comprehensiveness in highly complex physiological environments, those models have certain limitations in the respect of lncRNA characteristics descriptions. In order to get around this, we developed a novel model to calculate lncRNA functional similarity by integrating heterogeneous network data to predict lncRNA functional similarity effectively. This model included four single networks: miRNA-based similarity network described the lncRNA functional similarity in regulating the downstream RNAs; disease-based similarity network described the lncRNA functional similarity in the development of diseases; GTEx expression-based network and NONCODE expression-based network described the lncRNA expression similarity in each organ and tissue. Finally, the model integrated the data of four single lncRNA functional similarity networks with the Area Under ROC Curve (AUC) of each network for predicting lncRNA pairs with shared target mRNAs as the weights. On account of this integrated network completely considered these aspects described by four single networks, functional similarity values in this network were more comprehensive and accurate. Moreover, for facilitating users’ access, a web server named IHNLncSim was developed based on this mode, the modules of network visualization and disease-based lncRNA function enrichment analysis were also added into it.

## Materials and Methods

**FIGURE 1:**
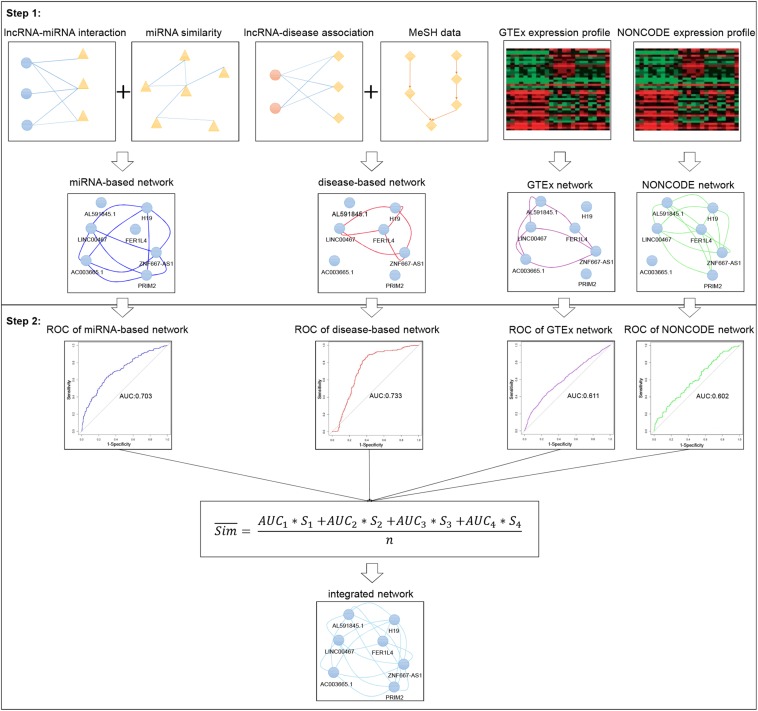
Flowchart of integrated network for predicting lncRNA functional similarity. In step 1, four separate lncRNA functional similarity networks were constructed using heterogeneous data sources, respectively. In step 2, the AUC values of the four networks were used as the weight, and the weighted average was taken as the final result of the integrated network.

In IHNLncSIm, four similarity network data about lncRNAs had been integrated to comprehensively analyze lncRNA features by using the AUC (for predicting lncRNAs with shared target mRNAs) of each network as a weight, the weighted averages of the similarity values of each network were taken as the final results of the integrated network (see Figure 1 for the workflow).

### MiRNA-Based lncRNA Similarity Network

**FIGURE 2:**
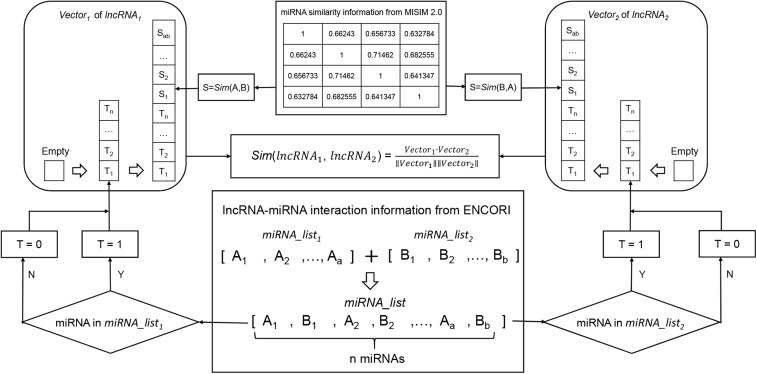
Flowchart of miRNA-based lncRNA similarity network. It demonstrates the basic ideas of calculating the functional similarity of two lncRNAs by using cosine correlation.

Prior knowledge had shown that lncRNAs with more common target miRNAs may have higher similarity (Tay et al., 2014). According to this assumption, the miRNA-based similarity network was developed by integrating lncRNA-miRNA interaction and miRNA functional similarity datasets (see Figure 2). Human lncRNA-miRNA interaction datasets were downloaded from ENCORI (Li et al., 2014)^1^ in January 2019. ENCORI is the updated version of StarBase database which provides the most comprehensive network of miRNA-lncRNA interactions supported by CLIP-Seq data sets. The collected data contains 9664 experimentally validated lncRNA-miRNA interactions which included 923 lncRNAs and 263 miRNAs. And we also collected human miRNA functional similarity data sets from MISIM v2.0 (Wang et al., 2010; Li et al., 2019)^2^ which is a bioinformatics tool not only for calculating human miRNA functional similarity, but also for predicting potential functions of miRNAs. Finally, 263 human miRNA functional similarities were obtained.

For example, in order to calculate the similarity of two interesting lncRNAs (*lncRNA*_1_ and *lncRNA*_2_), miRNAs interacted with the two lncRNAs are gathered into two aggregates (*miRNA_list_1_* and *miRNA_list_2_*). And the union between *miRNA_list_1_* and *miRNA_list_2_* are used to compile two miRNA vectors (*Vector*_1_ and *Vector*_2_) to represent *lncRNA*_1_ and *lncRNA*_2_, respectively (where 1 for presence of miRNA and 0 for absence). Next step, miRNA functional similarity values from MISIM, *Sim* (A, B) and *Sim* (B, A) were used to replace the values in *Vector*_1_ and *Vector*_2_.

After two feature vectors of the lncRNAs were constructed, the association score of *Vector*_1_ and *Vector*_2_ can be calculated by using cosine correlation as:

(1) $$S\,i\,m\,\left(l\,n\,c\,R\,N\,A_{1},l\,n\,c\,R\,N\,A_{2}\right)=\frac{V\,e\,c\,t\,o\,r_{1}\cdot V\,e\,c\,t\,o\,r_{2}}{||V\,e\,c\,t\,o\,r_{1}||\,||V\,e\,c\,t\,o\,r_{2}||}$$

### Disease-Based lncRNA Similarity Network

**FIGURE 3:**
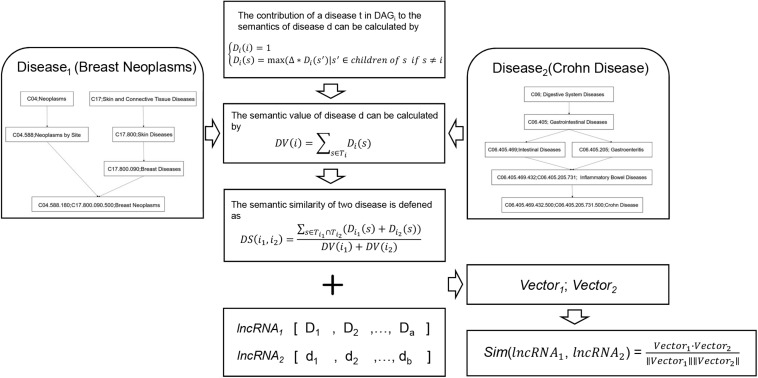
Flowchart of disease-based lncRNA similarity network. Firstly, disease semantic similarity among all the diseases was calculated based on the MeSH disease structure of DAG. Then, lncRNA semantic feature vector of each lncRNA was calculated. Finally, disease-based lncRNA similarity was calculated by using cosine correlation.

Based on the assumption that lncRNAs associated with similar diseases may have similar functions, lncRNA functional similarity can be calculated by integrating disease similarity and known lncRNA-disease association data sets, the flowchart of disease-based lncRNA similarity network is shown in Figure 3. In this network, we collected MeSH disease descriptors and MeSH disease structure of Directed Acyclic Graph (DAG) (Lipscomb, 2000) from the National Library of Medicine^3^ in January 2019 to build disease semantic similarity matrix. The MeSH database gave a universal disease classification system of diseases, and MeSH descriptors were divided into 16 categories. Among these categories, Category C for disease terms was extensive used in displaying the relationship between various diseases because of their DAG structures. In the DAG structures, each disease term was considered as a node that connected with parent node by a direct edge. Generally, one parent node showed a more general term and generalized the common attributes shared by its all child nodes, a child node showed a more specific term and was descripted as its parent node’s extensions in the DAG structures. In our study, human lncRNA-disease association datasets were downloaded from LncRNADisease v2.0^4^ (Bao et al., 2019), which was a high-quality database for studying lncRNA-disease associations and explored the potential function of lncRNA in a wide variety of diseases. After transforming disease names based on MeSH glossary, 2298 experimentally validated lncRNA-disease associations were curated, covering 454 lncRNAs and 271 diseases.

The construction process of disease-based lncRNA similarity network mainly includes the following steps. Firstly, semantic similarity between each pair of diseases should be built. Wang et al. (2010) provided a calculation method of disease semantic values. Based on the MeSH disease structure of directed acyclic graph (DAG), and the *DAG*_*i*_ = (*i*,*T*_*i*_,*E*_*i*_), where *i* is a disease in the DAG structure, *T*_*i*_ is a list including its all ancestor nodes and itself, *E*_*i*_ is another list of links between them. Defining the contribution of disease s in *DAG*_*i*_ named *D*_*i*_(*s*) as follow:

(2) $$\left\{ \begin{array}{c} D_{i}\,\left(i\right)=1\\ D_{i}\left(s\right)=max(\Delta *D_{i}\left(s^{\prime }\right)|s^{\prime }\in childrenofsifs\neq i \end{array}\right.$$

where △ is the decay factor of ancestor nodes’ semantic contribution which is set between 0 and 1, usually 0.5 is more suitable value. Obviously, the farther disease term *s* is from disease *i*, the lower semantic contribution *s* has. The most specific node *i* has the maximum semantic contribution 1. After this step, calculating semantic value of disease *i* named *DV*(*i*) as follow:

(3) $$D\,V\,\left(i\right)=\sum_{s\in T_{i}}D_{i}\,\left(s\right)$$

Directed acyclic graph can not only represent the disease term structure, but also offer two diseases relative position information. It is generally recognized that the more same or closer nodes two disease terms contain, the more similar they are. According to this theory, the semantic similarity of two diseases *i*_*1*_ and *i*_2_is defined as follow:

(4) $$D\,S\,\left(i_{1},i_{2}\right)=\frac{\sum_{s\in T_{i_{1}}\,\cap T_{i_{2}}}\left(D_{i_{1}}\,\left(s\right)+D_{i_{2}}\,\left(s\right)\right)}{D\,V\,\left(i_{1}\right)+D\,V\,\left(i_{2}\right)}$$

The semantic similarity matrix of diseases is gained in the end and it is convenient to fetch the semantic similarity between disease *i*_1_ and *i*_2_.

Secondly, based on the lncRNA-disease association from the datasets in LncRNADisease v2.0, the matrix of semantic feature of the lncRNA-disease association data is calculated. Each lncRNA-disease association can be quantified as semantic feature vector, which is defined as

(5) $$V\,e\,c\,t\,o\,r_{l\,n\,c\,d}=\left[D\,V\,\left(i_{d\,1}\right),D\,V\,\left(i_{d\,2}\right),\ldots ,D\,V\,\left(i_{d\,n}\right)\right]$$

where *n* is the total number of diseases associated with lncRNA *lncd*. The *Vector*_*lncd*_ represents the global semantic features of the diseases regulated by lncRNA *lncd* in the MeSH disease structure of DAG.

At last, the functional similarity of two interesting lncRNAs, their semantic feature vectors are *Vector*_*lncd*_ and *Vector*_*lnct*_, can be also calculated by using cosine correlation such as formula (1).

### GTEx Expression-Based Network and NONCODE Expression-Based Network

LncRNAs can be also characterized with expression profiles, they have relative lower expression level and much more tissue-specific pattern, which shows different expression level between different tissues, organs and growth stages (Harrow et al., 2012; Zhu et al., 2014). Prior knowledge has shown that co-expressed genes often share common functions (Eisen et al., 1998; Chen and Yan, 2013). Similarly, expression profiles of lncRNAs also implicate their functions, and thus measuring lncRNA functional similarity can be conducted through comparing their expression profiles (Chen et al., 2019). Therefore, We collected the expression profile datasets from GTEx v7 (The Genotype-Tissue Expression)^5^ (Consortium, 2013) and NONCODE v5.0^6^ (Fang et al., 2018), respectively. GTEx v7 project is a continuous effort to establish an integrated public database for the sake of studying tissue-specific gene expression and regulation. NONCODE v5.0 is a comprehensive annotation database, which contributes to non-coding RNA (excluding tRNA and rRNA). However, there were different criterions in evaluation of gene expression between two databases, GTEx used the TPM arithmetic and NONCODE adapted the FPKM. Moreover, lncRNA expression data from GTEx included 53 organs or tissues while NONCODE included 23, the two datasets cannot be combined simply. We constructed the similarity networks for these two datasets separately and acquired 4170 lncRNA expression profiles in GTEx v7 and 3073 lncRNA expression profiles in NONCODE v5.0. We defined those two similarity networks, respectively, and calculated their lncRNA expression similarity by utilizing the Spearman’s rank correlation coefficient analysis.

### Integrated Network Based on Above Heterogeneous Network Data

After above steps, four heterogeneous lncRNA functional similarity network data have been calculated successfully, but these similarities could not be simply added to describe the similarity between lncRNAs. The similarities from the integrating heterogeneous network must be comprehensive consideration. Therefore, a reasonable method to integrate these similarity network data was needed. In our study, AUC of each functional similarity network for discriminating the positive lncRNA pairs (with common target mRNAs) from the negative pairs (without common target mRNAs) was measured and exploited as the weight of its own similarity, and the weighted average schema, $\bar{S\,i\,m}$, was the similarity of the integrated network, which is defined as

(6) $$\bar{S\,i\,m}=\frac{A\,U\,C_{1}*S_{1}+A\,U\,C_{2}*S_{2}+A\,U\,C_{3}*S_{3}+A\,U\,C_{4}*S_{4}}{n}$$

where *n* is the number of integrating heterogeneous networks, *AUC*_*i*_ and *S*_*i*_ represent the weight and the similarity of network *i*.

### Server Construction

The framework of “Linux + Bootstrap + MySQL + Django” was adopted to construct the web server named IHNLncSim. The IHNLncSim is unrestricted (without a login procedure) and is compatible with most web browsers, it is accessible from http://www.lirmed.com/ihnlncsim. To facilitate the annotation, it also provided network visualization function which was implemented by open source visjs package^7^.

## Results

### lncRNA Functional Similarity Calculation

**FIGURE 4:**
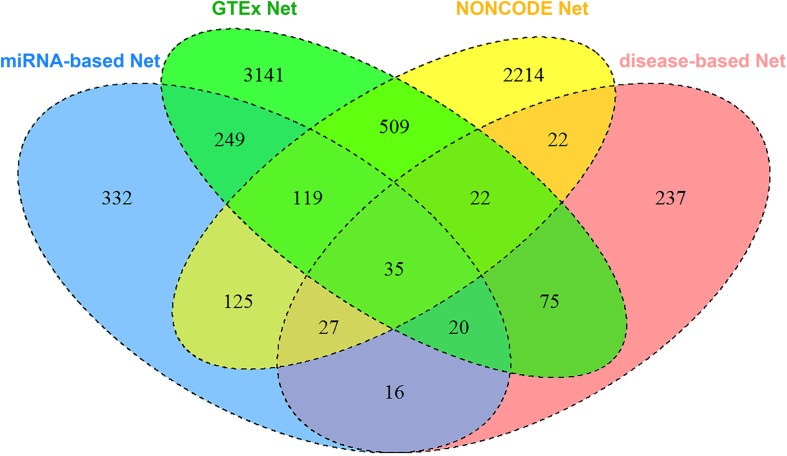
Venn diagram showing the overlap of lncRNAs between four functional similarity network data.

In total, we collected 7143 lncRNAs information from four heterogeneous data sources and calculated the similarity between two lncRNAs, respectively. Figure 4 shows the detailed lncRNA distribution in four functional similarity networks from IHNLncSim. Four single networks and the integrated network based on heterogeneous data sources were constructed. The lncRNA functional similarity values in each network were public in the download page of IHNLncSim to benefit the biological experimental validation^8^.

### Performance Evaluation of IHNLncSim

It had been shown that lncRNAs could regulate a number of key biological processes by pairing with their targeted RNAs, lncRNAs with common target genes will have higher functional similarity than lncRNAs without common target genes (Faghihi et al., 2008, 2010). Therefore, it is reasonable to measure lncRNA functional similarity by utilizing lncRNA-mRNA associations. In order to validate the performance of IHNLncSim, human lncRNA-mRNA interaction datasets were download from RISE^9^ (Gong et al., 2018). RISE was an excellent database of which contains abundant experimentally confirmed RNA interactome from sequencing experiments. After collecting and sorting data, 2462 experimentally verified lncRNA-mRNA interactions were gathered and 1312 sets of lncRNA pairs were constructed as positive samples, each set contained two lncRNAs sharing the same target mRNAs. It is noteworthy that each single similarity network did not cover all of these 1312 pairs. In our study, there are 273 positive samples pairs in the miRNA-based network, 323 pairs in the disease-based network, 850 pairs in the GTEx network, and 200 pairs in the NONCODE network. For constructing control sample pairs, we randomly selected a corresponding number of lncRNA pairs as negative samples in each network which had no shared target genes.

**FIGURE 5:**
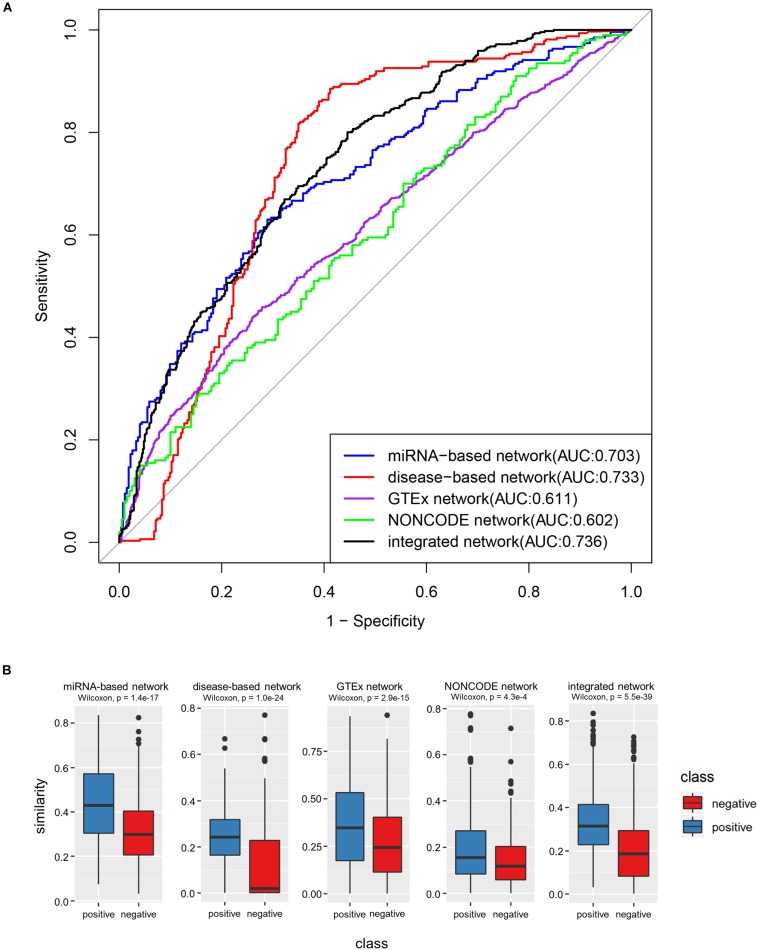
The performance of integrated similarity to discriminate lncRNA pairs with shared target mRNAs. **(A)** ROC curves of four single networks and integrated network. **(B)** Box-plot comparing the similarities among lncRNAs with shared target mRNAs (positive samples) and those without (negative samples). The *p*-values were derived via Wilcoxon test.

Firstly, we tested how each similarity network could be used to discriminate lncRNAs with shared target mRNAs. The discrimination accuracies were evaluated by the AUC, and the experimental results showed that the miRNA-based network obtained an AUC of 0.703, the disease-based network obtained an AUC of 0.733, the GTEx network obtained an AUC of 0.611, and the NONCODE network obtained an AUC of 0.602. These AUC values were further exploited as the weight to construct the integrated similarity network. Next, the integrated network was also tested in similar fashion. There was a large difference in sample size of each similarity network, to balance the impact of each similarity network on the integrated network, we selected the same numbers of positive and negative samples in each similarity network data during verification experiments. Statistics indicated that the NONCODE similarity network had the least numbers of positive and negative samples, the number of samples was only 200. Therefore, we also selected 200 positive samples and 200 negative in the other three network data. In the selection, lncRNA pairs with similarity values in the more networks had higher priority. After merging the same samples, we collected 466 comprehensive positive samples and 561 comprehensive negative samples in the end. The validation experimental results were shown in Figure 5, where integrated network showed an improved AUC value of 0.736.

The most intuitive plots in experimental results were box-plots of Figure 5 (*P*-value, Wilcoxon test). We can clearly see that the functional similarity values between the positive lncRNA pair samples were higher than that of negative samples on the whole, which was consistent with the previous theory. In addition, as can be seen from the ROC curves in the above figure, the integrated network showed relatively better result in following two reasons. Firstly, the AUC value of the integrated network was significantly larger than that of the miRNA-based network, GTEx network and NONCODE network, indicating that the integrated network can better distinguish between positive and negative samples in comparative experiments. Secondly, although the AUC value of the disease-based network was extremely closed to the integrated network, its ROC curve was much inferior than the ROC curve of integrated network when FPR was small, which shown that integrated network had better prediction accuracy when the false positive rate was required to be low.

**FIGURE 6:**
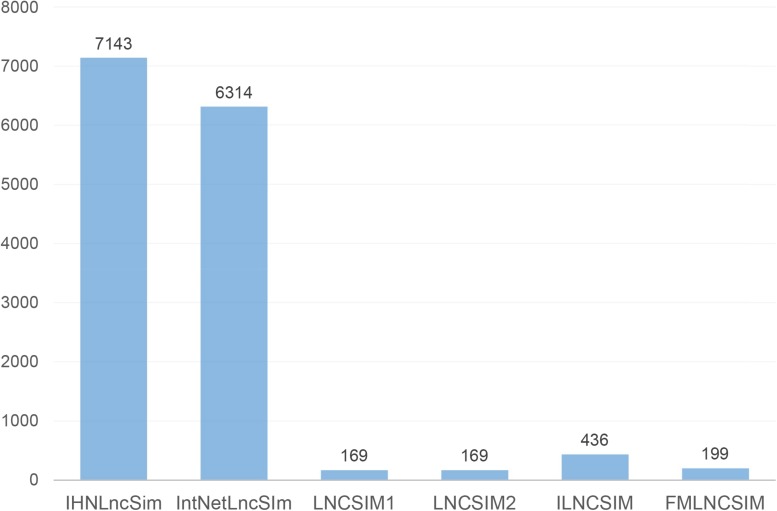
The number of lncRNAs in each lncRNA functional similarity calculation model.

**FIGURE 7:**
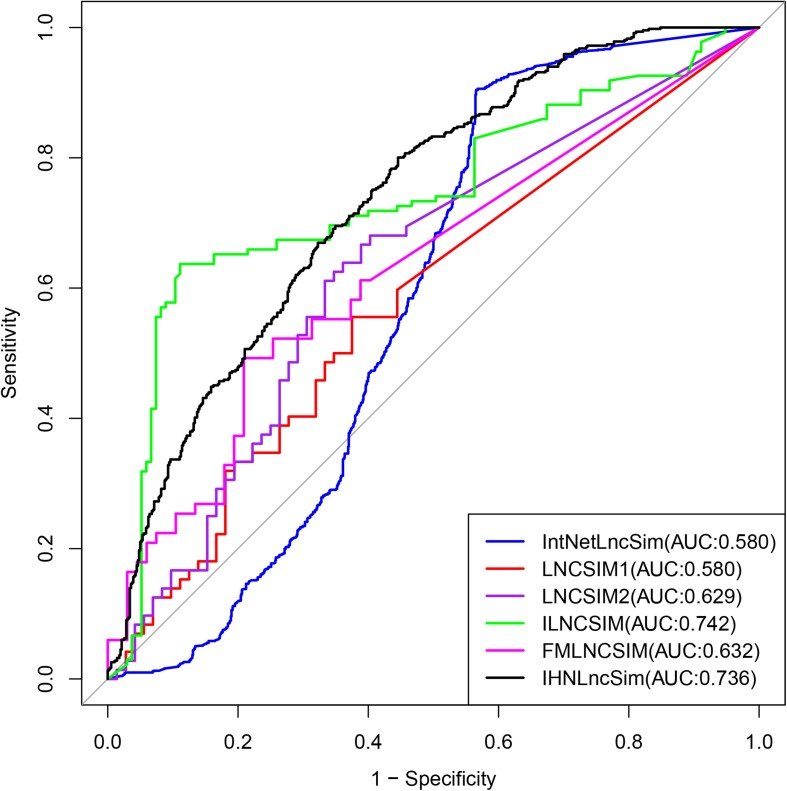
Performance evaluation for IHNLncSim and other existing lncRNA functional similarity calculation methods in terms of ROC curves and AUC.

In order to further evaluate the effectiveness of IHNLncSim, we compared its model with other existing lncRNA functional similarity calculation models. The similarity values of IntNetLncSim, LNCSIM1, LNCSIM2, ILNCSIM, and FMLNCSIM models can be obtained, respectively, through the corresponding web tools or data files. The number of lncRNAs in each model was shown in Figure 6. Limited by this, there were 1116 positive sample pairs in IntNetLncSim, 72 pairs in LNCSIM1, 72 pairs in LNCSIM2, 135 pairs in ILNCSIM, and 67 pairs in FMLNCSIM. Next step, negative sample pairs were randomly selected and verification experiments were performed as before. The validation experimental results were shown in Figure 7, where the IntNetLncSim obtained an AUC of 0.580, the LNCSIM1 obtained an AUC of 0.580, the LNCSIM2 obtained an AUC of 0.629, the ILNCSIM obtained an AUC of 0.742, and the FMLNCSIM obtained an AUC of 0.632. In the same experiment, the integrated network in IHNLncSim obtained an AUC of 0.736, which was higher than most of other models. Overall, it can be seen IHNLncSim performed well in lncRNA functional similarity calculation. Although the model of ILNCSIM obtained a comparable AUC of 0.742, the number of lncRNA similarity values included in ILNCSIM was much less than IHNLncSim. In general, after a series of comparative verification experiments, we can conclude that the lncRNA similarity model constructed in this paper is reliable, IHNLncSim would be further improved when more data could be obtained in the future.

### Overview of IHNLncSim Web Server

In IHNLncSim, the lncRNA functional similarity analysis module provided two input methods to users. One is “all vs. all,” users can submit a lncRNA list. The tool will analyze the functional similarity between lncRNAs in the input list. The other is “one vs. all,” and users just submit one lncRNA name each time. IHNLncSim will analyze the functional similarities between this lncRNA and one of all lncRNAs from four heterogeneous network data. For the convenience of users, five types of lncRNA names were allowed users to submit in the IHNLncSim, which included gene symbol and alias, Ensembl ID, RefSeq ID, NONCODE ID and chromosomal location. If users were interested in our data, the download page provided the function to download the calculated results.

The function for result visualization is enabled in the IHNLncSim to facilitate the community. The nodes in the network diagram represent lncRNAs, and the color of edges represent the class of similarity network. Nodes can be freely dragged to change the network layout by mouse. Moreover, users can also click on the edges to show the values of functional similarity between two lncRNAs with common links. The networks could also be downloaded as text files, which contained all similarities between two lncRNAs. Once the networks were finalized, users also could download the network pictures.

### Example of Analyzing Functional Similarity for a List of lncRNAs

**FIGURE 8:**
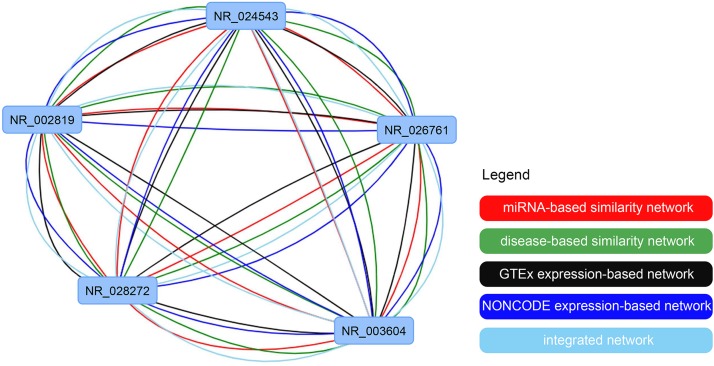
The visualization of the constructed lncRNA functional similarity networks using four single similarity networks and the integrated similarity network.

To show the lncRNA similarity calculate function of IHNLncSim, we choose one from lncRNA sample lists in “all vs. all” menu as an example and then submit it to IHNLncSim. After analyzing by IHNLncSim, the lncRNA functional similarity values of four single networks and the integrated network are shown in a table in the center of the page. Next, we can download the analyzed results to a text file or visualize them as a network. At last, user can adjust the position, size and shape of the nodes for a clear and nice-looking effect. The visualization of all networks in this example is shown as follow (see Figure 8).

## Discussion

Computation of functional similarity between lncRNAs shows great significance in the study of the unknown function of lncRNAs. Fortunately, a various lncRNA-related biological databases have made it feasible to develop computational models for predicting lncRNA functional similarities. Here, we present a novel model that used heterogeneous network data which cover the miRNA interactions, disease associations and expression features of the lncRNAs. A web server named IHNLncSim was constructed, it not only can calculate the functional similarities with respect to the query lncRNAs but also can implement the visualization of lncRNA functional similarity networks.

Of course, there are also some limitations existing in IHNLncSim. One limitation is that the lncRNA-miRNA interaction and lncRNA-disease association data are still incomplete, which could produce bias when calculating miRNA-based lncRNA functional similarity and disease-based lncRNA functional similarity. With the growing of related biomedical data, the similarity calculation of integrated network based on heterogeneous network data would be further improved. Another limitation is that the visualization of lncRNA functional similarity networks fails to run properly for some internet browsers. We will continue testing IHNLncSim in different internet browsers to make it gain multi-browser support. The third limitation is that currently IHNLncSim only runs for human but cannot be applied to other species, we will collect the biological data from other species, for example, mouse and rat, and update IHNLncSim as well in the future.

## Data Availability Statement

Publicly available datasets were analyzed in this manuscript. Human miRNA functional similarity datasets can be found in the MISIM v2.0 (http://www.lirmed.com/misim/). MeSH datasets can be found in the National Library of Medicine (http://www.nlm.nih.gov/). Human lncRNA-disease association datasets can be found in the LncRNADisease v2.0 (http://www.rnanut.net/lncrnadisease/). Human expression profile datasets can be found in the GTEx v7 (https://www.gtexportal.org/) and NONCODE v5.0 (http://www.noncode.org/). Human lncRNA-mRNA interaction datasets can be found in the RISE (http://rise.life.tsinghua.edu.cn/).

## Author Contributions

JL and LL designed the study. YiZ and SZ performed the analysis. YiZ drafted the manuscript. JL, YuZ, and LL revised the manuscript.

## Conflict of Interest

The authors declare that the research was conducted in the absence of any commercial or financial relationships that could be construed as a potential conflict of interest.

## Footnotes

1 http://starbase.sysu.edu.cn/

2 http://www.lirmed.com/misim/

3 http://www.nlm.nih.gov/

4 http://www.rnanut.net/lncrnadisease/

5 https://www.gtexportal.org/

6 http://www.noncode.org/

7 https://visjs.org/

8 http://www.lirmed.com/ihnlncsim/download/

9 http://rise.life.tsinghua.edu.cn/
